# Genomic divergence between Spanish *Littorina saxatilis* ecotypes unravels limited admixture and extensive parallelism associated with population history

**DOI:** 10.1002/ece3.4304

**Published:** 2018-07-23

**Authors:** Tony Kess, Juan Galindo, Elizabeth G. Boulding

**Affiliations:** ^1^ Department of Integrative Biology University of Guelph Guelph ON Canada; ^2^ Departamento de Bioquímica Genética e Inmunología Facultad de Biología Universidade de Vigo Vigo Spain; ^3^ Centro de Investigación Mariña da Universidade de Vigo (CIM‐UVIGO) Vigo Spain

**Keywords:** ecological speciation, genomics, local adaptation, marine snail, parallelism, RAD sequencing

## Abstract

The rough periwinkle, *Littorina saxatilis*, is a model system for studying parallel ecological speciation in microparapatry. Phenotypically parallel wave‐adapted and crab‐adapted ecotypes that hybridize within the middle shore are replicated along the northwestern coast of Spain and have likely arisen from two separate glacial refugia. We tested whether greater geographic separation corresponding to reduced opportunity for contemporary or historical gene flow between parallel ecotypes resulted in less parallel genomic divergence. We sequenced double‐digested restriction‐associated DNA (ddRAD) libraries from individual snails from upper, mid, and low intertidal levels of three separate sites colonized from two separate refugia. Outlier analysis of 4256 SNP markers identified 34.4% sharing of divergent loci between two geographically close sites; however, these sites each shared only 9.9%–15.1% of their divergent loci with a third more‐distant site. STRUCTURE analysis revealed that genotypes from only three of 166 phenotypically intermediate mid‐shore individuals appeared to result from recent hybridization, suggesting that hybrids cannot be reliably identified using shell traits. Hierarchical AMOVA indicated that the primary source of genomic differentiation was geographic separation, but also revealed greater similarity of the same ecotype across the two geographically close sites than previously estimated with dominant markers. These results from a model system for ecological speciation suggest that genomic parallelism is affected by the opportunity for historical or contemporary gene flow between populations.

## 1 INTRODUCTION

Strong evidence for a primary role for natural selection in driving ecological speciation is found in cases in which similar local selective forces generate comparably adapted phenotypes and similar sources of reproductive isolation (Arendt & Reznick, 2008; Rundle & Nosil, 2005; Schluter & Nagel, 1995). Although the connection between parallel adaptation, isolation, and selection is straightforward at the phenotypic level, the frequency that these traits arise through parallel genomic mechanisms is less clear, representing an important challenge in the field of speciation genomics (Hoban et al., 2016; Seehausen et al., 2014; Wolf & Ellegren, 2017). One of the key directions in this research is in uncovering the extent of genetic parallelism and elucidating different processes that could produce varying degrees of this parallelism (Conte, Arnegard, Peichel, & Schluter, 2012; Elmer & Meyer, 2011; Nosil, 2012).

The extent of geographic and genetic separation between populations undergoing parallel adaptation may play a role in determining observed levels of shared genomic differentiation (Conte et al., 2012; Johannesson et al., 2010; Ord & Summers, 2015). Populations that show greater connectivity or a recent common origin are likely to share similar complements of standing genetic variation that may be included in the genetic architecture of ecologically adaptive traits (Barrett & Schluter, 2008). Connected or recently separated populations are also expected to share similar genetic backgrounds that determine constraints on adaptive *de novo* mutations (Conte et al., 2012). Additionally, migration between populations will dictate the likelihood that ecologically advantageous mutations will be transmitted and selected across sites (Johannesson et al., 2010; Ralph & Coop, 2010; Rosenblum, Parent, & Brandt, 2014).

Adoption of next‐generation sequencing methods (NGS) in studies of ecological adaptation has allowed simultaneous identification of genome‐wide distributions of locally selected loci and high‐resolution estimates of population genetic structure (Bernatchez, 2016; Bonin, Ehrich, & Manel, 2007; Hohenlohe et al., 2010). Genome scans, which detect outlier regions of elevated genomic divergence that may reflect reduced gene flow due to adaptation (Nosil et al., 2009), can be conducted across replicate instances of ecological divergence to quantify genomic parallelism through identifying proportions of outlier sharing (Bernatchez, 2016; Lewontin & Krakauer, 1973; Westram, Panova, Galindo, & Butlin, 2016). However, shared ancestry or contemporary migration between populations may constrain the genetic independence of parallel phenotypic evolution. Observed levels of genomic parallelism can be contextualized by quantifying population genetic structure and phylogenetic distance between instances of parallel adaptation, to determine whether rates of genomic parallelism are elevated in more closely related populations (Lowe & Allendorf, 2010; Pritchard, Stephens, & Donnelly, 2000).

Next‐generation sequencing studies in model species for ecological speciation have revealed substantial variation in genomic parallelism between systems, inferred from the proportion of shared genomic outlier regions exhibiting divergence beyond a neutral threshold. Investigation of shared parallel divergence in stick insect ecotypes (*Timema cristinae)* (Soria‐Carrasco et al., 2014) has revealed modest (17%) sharing of genomic divergence between ecotype pairs across multiple populations, whereas comparisons of parallel genomic divergence in freshwater and marine stickleback (*Gasterosteus aculeatus*) ecotypes have uncovered greater (35%) genomic reuse (Jones et al., 2012). A meta‐analysis of rates of parallel molecular evolution from a small sample of studies that passed inclusion criteria, conducted by Conte et al. (2012), identified substantial reuse of individual candidate genes (55%) or genomic regions (32%) associated with a selected trait. This rate of genomic parallelism declined with phylogenetic distance between compared groups, suggesting that the extent of evolutionary independence between lineages is important to how repeated molecular evolution occurs. Given the great heterogeneity of observed patterns, identification of the evolutionary and ecological contexts in which parallel genomic differentiation occurs across systems is a vital next step in understanding the genomics of parallel evolution.

**Figure 1:**
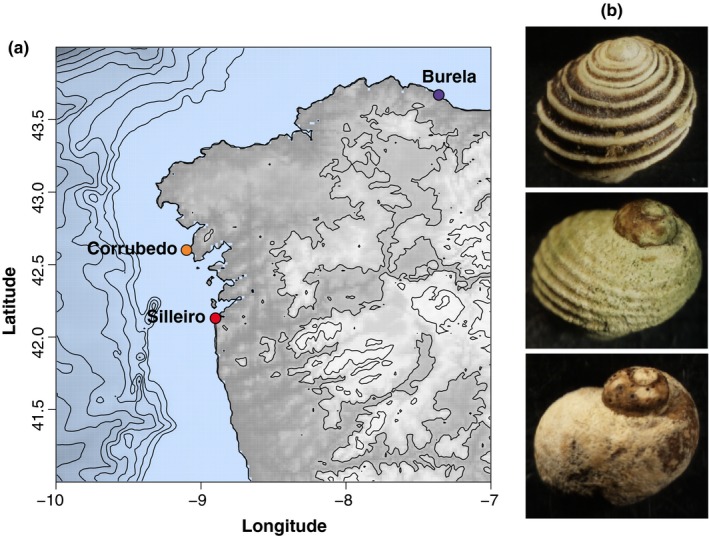
(a) Sampling site locations on Galician coast of Spain. (b) Representative ecotype shell patterns: from top to bottom, ridged and banded (crab ecotype), ridged unbanded (intermediate), smooth unbanded (wave ecotype)

To investigate the genomics of parallel ecological speciation with gene flow, we studied genome‐wide divergence and population structure of *Littorina saxatilis* ecotype pairs from different tidal levels and sites on the Galician coast of Spain (Figure 1). These marine gastropods form ecotype pairs in replicate across several shores in the UK, Spain, and Sweden, adapted to either wave action or crab predation across rocky intertidal zones in the northeast Atlantic and are differentiated by life history, behavior, and shell morphology and ornamentation (Rolán‐Alvarez, Austin, & Boulding, 2015; Galindo & Grahame, 2014; Johannesson et al., 2010; Rolán‐Alvarez, 2007; Rolán‐Alvarez, Johannesson, & Erlandsson, 1997). These ecotypes are considered a model system for the study of parallel incipient ecological speciation with gene flow (Nosil, 2012; Schluter, 2009), as local adaptation and subsequent extrinsic reproductive isolation are believed to have evolved independently in response to similar ecological pressures across multiple populations, despite molecular evidence for gene flow between ecotypes (Butlin et al., 2014; Johannesson et al. 1995; Johannesson et al., 2010; Quesada, Posada, Caballero, Morán, & Rolán‐Alvarez, 2007; Rolán‐Alvarez et al., 2004). Although gene flow is still ongoing (Butlin et al., 2014; Galindo, Martínez‐Fernández, Rodríguez‐Ramilo, & Rolán‐Alvarez, 2013), partial barriers to gene flow have evolved through divergent selection on shell and foot size and morphology, life history, habitat choice, and size‐assortative mating (Cruz et al., 2004; Boulding et al., 2017; Galindo & Grahame, 2014; Rolán‐Alvarez, 2007; Rolán‐Alvarez et al., 2015).

Previous measures of population genetic structure of Spanish ecotypes using microsatellites, neutral allozymes, and AFLP loci have indicated greater divergence between separate sites than between ecotypes at different tidal levels on the same shore, providing support for the hypothesis that evolution of ecotypes has occurred in microparapatry with minimal spatial separation to prevent gene flow (Galindo, Morán, & Rolán‐Alvarez, 2009; Rolán‐Alvarez et al., 2004). Mitochondrial phylogenetic analysis of crab and wave ecotypes across the full Spanish geographic range has revealed mtDNA haplotype trees that cluster by geography rather than ecotype, indicating that neutral sequence divergence has accumulated between sites isolated by distance, rather than between ecotypes (Quesada et al., 2007). Comparison of different historical demographic hypotheses supports origin of northern and southern populations of Spanish ecotypes from two separate glacial refugia (Butlin et al., 2014), consistent with previous phylogeographic studies (Doellman, Trussell, Grahame, & Vollmer, 2011; Panova et al., 2011).

Surprisingly, phylogenetic or geographic distance has not yet been shown to affect rates of genomic parallelism across *L. saxatilis* ecotype pairs. Transcriptomic comparison of parallel divergence across the European range of *L. saxatilis* ecotypes found only moderate genomic parallelism between Swedish, UK and Spanish populations (Westram et al., 2014) despite the expectation that UK and Swedish sites should show greater similarity due to colonization from shared glacial refugia (Panova et al., 2011). Analysis of RAD‐derived SNP markers in Swedish ecotypes found at multiple sites within a single archipelago at short geographic distance (10 km) identified low‐to‐moderate (8%–28%) genomic parallelism between sites despite restriction of the spatial scale of comparisons (Ravinet et al., 2016). Thus, *L. saxatilis* ecotypes appear to defy the expectation that reduced separation corresponds to greater sharing of genomic divergence. A genome‐wide study including sites at varying levels of geographic and evolutionary divergence has yet to be carried out to clarify the role of connectivity in dictating genomic parallelism.

Here, we study patterns of genomic divergence and parallelism in Spanish *L. saxatilis* wave and crab ecotypes and putative hybrids of intermediate phenotype in three geographically separated sites. We test the hypothesis that the degree of genomic parallelism associated with adaptation to replicated ecological conditions on the upper or lower shore is affected by the possibility of historical or contemporary gene flow between sites and will show correspondence with geographic separation between sites. We predict that samples from the same tidal level at geographically closer sites that shared a glacial refuge will exhibit less neutral genetic differentiation and will also demonstrate greater levels of shared genomic divergence than more distant sites. We used a recently developed, inexpensive double‐digest RAD sequencing protocol (Kess, Gross, Harper, & Boulding, 2016) to quantify population structure, phylogenetic separation, and parallel genomic divergence between crab and wave ecotype snails from three sites that have colonized intertidal zones from two glacial refugia. Consistent with our prediction, we find levels of genomic parallelism correspond to the degree of geographic separation. Surprisingly, we identify greater genomic differentiation between tidal levels of the same site than has previously been revealed using neutral molecular markers, and find little evidence of admixture in phenotypically intermediate individuals from the mid‐shore. This study contributes to our understanding of factors dictating genomic parallelism and provides new evidence of more extensive genomic isolation and progress toward potential microparapatric speciation in the model “crab” and “wave” ecotypes of *L. saxatilis*.

## 2 METHODS

### 2.1 Sampling design and study sites

Samples were obtained from three sites across the known distribution of crab and wave *L. saxatilis* ecotypes in NW Spain, depicted in Figure 1a: Silleiro (Long – 8.90°, Lat 42.13°), Corrubedo (Long – 9.10°, Lat 42.60°), and Burela (Long – 7.36°, Lat 43.67°). These three sites allow comparison of population genetic structure and genomic divergence between ecotypes from two separate postglacial colonizations, as recent evidence suggests Burela and Silleiro have been colonized from distinct northern and southern refugia (Butlin et al., 2014; Tirado, Saura, Rolán‐Alvarez, & Quesada, 2016).

We sampled two parallel transects in each site, (Silleiro, 45 m apart; Corrubedo, 10 m; Burela, 25 m) and within each transect three tidal levels: upper shore (predominantly crab ecotype, above the upper limit of the barnacle zone), mid‐shore (patchy habitat of mussels and barnacles with overlap of crab and wave ecotypes and frequent intermediate phenotypes), and lower shore (predominantly wave ecotype, at the lower limit of the mussel bed). Within the upper and lower shore levels, ~50 individuals were randomly sampled from each transect. Within the mid‐shore, ~100 phenotypically intermediate individuals were sampled from each transect, avoiding individuals with phenotypes characteristic of ecotypes in the upper and lower shore. Individuals with intermediate phenotype were categorized by the presence of only one of two diagnostic shell characteristics, shell bands or ridges, or intermediate combinations of these phenotypes. These diagnostic characters have previously been used to identify individuals likely arising from recent hybridization between ecotypes (Galindo et al., 2013; Johannesson, Johannesson, & Rolán‐Alvarez, 1993). By focusing on phenotypic intermediates, we intended to increase the chances of sampling admixed genotypes formed by hybridization between the two ecotypes. We froze all sampled individuals at −80°C and stored them in a −20°C freezer until DNA extraction.

### 2.2 DNA extraction

We selected 32 individuals randomly from each sampled tidal level (upper, mid‐shore, and lower) from each transect for DNA extraction. We separated body tissue from the shell using forceps and then further dissected individuals into foot and visceral mass sections. Foot tissue was flash‐frozen in liquid nitrogen and then ground in a mortar and pestle. Ground tissue was then extracted using E.Z.N.A Mollusc DNA kits (Omega Bio‐Tek). DNA quality was visualized on 2% TAE agarose gel against a 100‐bp DNA ladder (Invitrogen) and checked for concentration and purity on a NanoDrop spectrophotometer (Thermo Fisher, Inc.).

### 2.3 Library construction and sequencing

Libraries were constructed using the protocol described in Kess et al. (2016), using *PstI* and *BglII* restriction enzymes in a double digest of ~1,125 ng of genomic DNA. Enzymes were selected based on frequency of digestion and the absence of either *PstI* or *BglII* cut site sequence within repetitive element sequence identified in *L. saxatilis* ecotypes by Wood, Grahame, Humphray, Rogers, and Butlin (2008).

Adapter sequences and annealing and ligation conditions were unchanged from the previously described protocol. PCR amplification of adapter‐ligated product and addition of S50X and N70X Illumina adapters was also completed according to the previous protocol using Illumina indexes designed from Nextera XT Index v2 sequences. The same set of indexes were used for each of the three sampled locations; individuals from each site were sequenced on a separate lane of an Illumina Hiseq, to prevent sample mix‐up when demultiplexing.

A total of 541 individuals produced PCR products that could be visualized on a 2% TAE agarose gel. From each dual‐indexed sample, 10 uL of PCR product was pooled in a 1.5‐mL tube with PCR products from other sampled individuals from the same tidal level and site, creating groups of twelve individuals per pool in a final volume of 120 μl. Each tube was concentrated in a SpeedVac for 15 min to a final volume of 60 μl and then size selected in the 300‐ to 600‐bp size range using a Pippin Prep gel cassette system (Sage Biosciences) at the London Regional Genomics Centre in London, Ontario. Samples were then combined in three libraries with a final volume ≥20 μl and a concentration ≤5 ng/μl, with each library representing a different sampled site (Burela, Corrubedo, Silleiro). Libraries were then sequenced using paired‐end, 125‐bp sequencing reagent kits on an Illumina HiSeq 2500, at the Clinical Genomics Centre (CGC) in Toronto, Ontario. Sequences were then demultiplexed at the CGC and attributed to a sequenced individual using Hiseq N7 and S5 indexes.

### 2.4 Sequence quality analysis and control

Read number, base pairs sequenced, *Q* quality scores per lane and per sequenced individual, average and per base quality score, base content, and adapter content were assessed at the CGC using FastQC 0.11.0 (Andrews, 2014). We conducted all subsequent sequence quality checks and bioinformatic analyses using the Shared Hierarchical Academic Research Computing Network (SHARCNET) Linux cluster. Sequenced adapter content adjacent to restriction fragment inserts was identified at the end of several sequenced DNA fragments. We trimmed sequenced fragments for quality by removing regions of bases that fell below a *Q* score of 20 within a 10‐bp sliding window and removed adapter and index material using Trimmomatic v 0.32 (Bolger, Lohse, & Usadel, 2014). A second round of quality analysis was carried out using the *process_radtags* module in Stacks 1.40 (Catchen, Amores, Hohenlohe, Cresko, & Postlethwait, 2011) to remove any sequenced fragments with uncalled bases or ambiguous restriction enzyme sequences at the beginning of forward or reverse reads. All reads were truncated to a length of 85 bp, and reads falling below this read length threshold were discarded. The *rescue_radtags* option was enabled to retain reads with restriction enzyme cut sequences with individual base mismatch deviations from the expected cut site sequence.

### 2.5 De novo assembly, SNP identification, and validation

We used Stacks 1.40 to identify unique genomic fragments sampled by double restriction enzyme digestion (RAD loci) and SNPs within these fragments. We retained an average of 683,330 reads per individual and used only individuals with a minimum of 160,000 high‐quality reads that passed both quality filtering steps for SNP genotyping. We used *denovo_map.pl* in Stacks to identify distinct RAD loci and SNPs within individuals (*ustacks*), to build a consensus catalog of RAD and SNPs within sampled ecotypes and sites (*cstacks*) and to match individually identified loci against the catalog of consensus loci (*sstacks)*. To identify polymorphic loci, we set minimum coverage of five reads per sampled RAD locus in an individual and allowed a RAD locus read to mismatch by up to 8 bp between or within individuals. These Stacks parameters are consistent with mismatch values determined by Ravinet et al. (2016) to account for high levels of heterozygosity encountered within the *L. saxatilis* genome. After genotyping, we used the *populations* module to output genotypes passing a minimum threshold of 70% individuals genotyped and minor allele frequency >1%. We selected only the first SNP from each RAD site to minimize short‐range physical linkage between SNPs on the same RAD locus that could bias population structure analyses. To quantify possible erroneously identified SNPs, we used VCFtools (Danecek et al., 2011) to carry out an exact test of Hardy–Weinberg Equilibrium (HWE) deviation in each tidal level and site (Wigginton, Cutler, & Abecasis, 2005) with a Bonferroni‐corrected α value of 5%, divided by the number of SNPs tested per grouping. Of all loci tested, we found that <5% of loci demonstrated genotypic frequencies that deviated from HWE. These loci were retained for outlier analysis as biological explanations for departure from HWE in a small subset of loci cannot be ruled out and may reflect genomic regions with distinct patterns of admixture rather than error in genotyping or SNP identification (Nosil, Parchman, Feder, & Gompert, 2012). We then used PGDSpider v 2.1.0.3 Lischer and Excoffier (2012) and VCFtools, and the *genepopedit* R package (Stanley, Jeffery, Wringe, DiBacco, & Bradbury, 2017) to convert Stacks output files to formats required by software used for outlier analysis, calculation of population structure, and phylogenetic comparison.

### 2.6 Outlier analysis

Detection of SNPs potentially under selection was carried out through three outlier detection methods. We conducted *F*_ST_ outlier analysis in ARLEQUIN v 3.5.2.1 (Excoffier & Lischer, 2010) implementing the standard FDIST method described by Beaumont and Nichols (1996). We also identified *F*_ST_ outliers using BayeScan 2.1 (Foll & Gaggiotti, 2008) and the *pcadapt* R package (Luu, Bazin, & Blum, 2017). We conducted outlier analysis between upper and lower shore sampled individuals in each site separately using each method. For the FDIST analysis, we carried out 20,000 simulations of 100 demes to build the null distribution for outlier identification. Following outlier analysis, we applied Sequential Goodness of Fit (SGoF) correction at 5% significance to reduce the possibility of spurious positive detection of outlier loci (Carvajal‐Rodríguez & de Uña‐Alvarez, 2011). BayeScan analysis was conducted with a sample size of 5000, running 20 pilot runs with 5,000 iterations each, followed by 10 thinning interval runs, each with 5,000 iterations, and burn‐in of 100,000 iterations. Loci identified in BayeScan were considered significant using 5% false discovery rate (FDR) cutoffs. Outlier detection in *pcadapt* was conducted retaining loci correlated with the first principal component axis after 5% FDR correction. The first principal component axis was selected through inspection of Scree plots for each site that indicated all subsequent axes explained only random variation (Luu et al., 2017). To account for false‐positive identification of outliers using individual outlier detection methods, we retained loci identified using at least two outlier methods for subsequent analysis. To test significance of shared outlier proportions between sites, we calculated 95% confidence intervals for the proportion of loci shared between two or three populations from randomly resampled lists of loci, as conducted by Soria‐Carrasco et al. (2014). Functional annotation of outlier RAD loci (85 bp in length) that showed evidence of divergent selection was performed using the BLASTX tool in the NCBI protein database.

### 2.7 Linkage disequilibrium

To identify potentially physically linked SNPs, we calculated pairwise linkage disequilibrium of all 4,256 loci within each site and shore level using plink v1.90b3.46 (Chang et al., 2015; Purcell et al., 2007). Because demographic and evolutionary processes can generate statistical linkage, we categorized potentially physically linked loci as those exhibiting *r* ^2^ above 0.2 in all sites.

### 2.8 Population structure and admixture

We used the Bayesian clustering program STRUCTURE v2.3.4 (Pritchard et al., 2000) to detect population structure and admixture between ecotypes from each sampled tidal level and site. We conducted separate analyses for the total set of 4,256 loci that passed quality filtering, 193 divergent loci, and 2,549 neutrally evolving loci that did not exhibit patterns of differentiation consistent with divergent or balancing selection in any comparison. STRUCTURE analyses were conducted using the *parallelstructure* R package (Besnier & Glover, 2013), with three replicates per *K* value for *K* = 1 to *K* = 9, with 100,000 burn‐in steps and 500,000 MCMC sampling steps. We visualized results in STRUCTURE HARVESTER (Earl & vonHoldt, 2012) to identify the value of *K* with highest Δ*K* (Evanno, Regnaut, & Goudet, 2005). To quantify admixture within and among ecotypes from separate sites, we investigated patterns of population structure of neutral loci for *K* = 6. Replicate runs for the *K* value chosen for each set of loci were summarized and graphically displayed using CLUMPAK (Kopelman, Mayzel, Jakobsson, Rosenberg, & Mayrose, 2015). To visualize population structure and provide secondary support for STRUCTURE comparisons, we then conducted discriminant analysis of principal components (DAPC) and principal component analysis (PCA) for divergent and neutral sets of loci. These analyses were conducted in the *adegenet* 2.0.0 R package (Jombart, 2008; Jombart & Ahmed, 2011).

### 2.9 Genetic differentiation

We used hierarchical analysis of molecular variance (AMOVA) (Excoffier, Smouse, & Quattro, 1992) implemented in ARLEQUIN to identify the extent of population structure caused by separation between tidal levels (upper and lower shore) and sites. We included only upper and lower shore individuals in all AMOVA tests. For each set of loci (all loci, neutral, and divergent), we grouped individuals by sampled tidal level or site. We then calculated *F*_CT_ and *F*_SC_ values to quantify differentiation driven by geographic separation between sites or ecotype divergence between tidal levels at different levels of hierarchical grouping. We conducted two analyses, one including all localities, and one analysis characterizing genetic differentiation between the two southern sites (Corrubedo and Silleiro), to account for potential genetic differentiation driven by the distant Burela site. Due to different sample sizes of genotyped individuals at each locus, we performed locus‐by‐locus AMOVA averaged over all loci with 1,000 permutations to calculate *F* statistic significance. Pairwise *F*_ST_ was calculated between all transects, tidal levels, and sites for all neutral and divergent loci in ARLEQUIN, using 1,000 simulations to assess significance.

### 2.10 Phylogenetic analysis

We calculated Cavalli‐Sforza and Edwards' chord distances (1967) between all individuals separately for 2,000 randomly selected neutral loci and all 193 divergent outlier loci in POPULATIONS 1.2.33 (Langella, 1999). Neighbor‐joining (NJ) trees were calculated with 1,000 bootstrap replicates on all loci. Trees were visualized with midpoint‐rooting in FigTree v1.4 (http://tree.bio.ed.ac.uk/software/figtree/).

## 3 RESULTS

### 3.1 Sequence quality analysis and control

We sequenced 541 individuals (173 Burela, 178 Corrubedo, 190 Silleiro), producing 697,554,454 total reads, of which 82% (577,932,750 total reads) could be assigned to a sequenced individual. An average of 1,067,720 reads could be assigned to each sequenced individual, with standard deviation of 730,224 reads. Read quality was uniformly high across all sequenced individuals, with an average *Q* score of 30 and standard deviation of 2.024. Following two rounds of quality filtering, we retained 477 sequenced individuals (158 Burela, 152 Corrubedo, and 167 Silleiro) with a minimum of 160,000 retained reads, and an average of 683,330 reads. Reads from these individuals were used in subsequent identification of RAD loci and SNPs.

### 3.2 SNP identification

We identified 127,476 unique RAD loci across 477 sequenced individuals, and 300,874 SNP sites were variable within those loci. Coverage per locus was high, with an average value of 20.55 reads and standard deviation of 6.50 reads per RAD locus across samples. We obtained a set of 4,256 filtered SNPs present in a minimum of 70% of samples, which we used to assess signatures of selection and population genetic structure.

### 3.3 Outlier analysis

We uncovered 240 unique loci demonstrating elevated patterns of divergence across all comparisons at 5% significance in FDIST, compared to only 59 unique outlier loci identified at 5% significance in BayeScan (Table 1). All significant loci identified by BayeScan were also identified using FDIST. Using *pcadapt*, we identified 275 loci that exhibited signals of potential selection and produced separation between ecotype clusters. Of these loci, 193 exhibited overlap with outliers identified using FDIST. A total of 1,098 loci at 5% significance exhibited low differentiation consistent with potential balancing selection occurring within sites using the FDIST method, whereas no loci under balancing selection were detected using BayeScan or *pcadapt*. BLASTX annotated seven outlier loci when the search was restricted to “Mollusca” and had an e‐value cutoff of 10^−8^ (Table 2).

**Table 1:** Number of polymorphic SNP loci compared in *F*_ST_ outlier tests between tidal levels in each site, percent, and total number of divergent outlier loci identified using FDIST significant at 5% in each site after SGoF correction and significant at 5% in BayeScan and pcadapt after false discovery rate correction, and average *F*_ST_ for neutral and divergent outlier loci in each site

Site	Polymorphic SNP loci	Divergent outliers significant at 5%			Average *F*_ST_ neutral loci	Average *F*_ST_ outlier loci
		FDIST	BayeScan	pcadapt		
Burela	2864	4.4% (125)	1% (28)	4.3% (123)	0.123	0.637
Corrubedo	2876	3.6% (105)	0.9% (27)	5.1% (146)	0.235	0.697
Silleiro	2507	3.5% (89)	1.1% (27)	3.3% (85)	0.152	0.744

**Table 2:** BLASTx hits of divergent outlier loci against the NCBI protein database within the phylum Mollusca

Outlier locus	BLASTx hit (Mollusca)	*e*‐value	Accession	Species
347	Uncharacterized protein LOC110449946	4.00E‐09	XP_021352804.1	*Mizuhopecten yessoensis*
783	PREDICTED: Spondin‐1‐like	7.00E‐11	XP_012941542.1	*Aplysia californica*
1650	PREDICTED: Mannosylglucosyl‐3‐phosphoglycerate phosphatase‐like	7.00E‐09	XP_014777376.1	*Octopus bimaculoides*
2097	Dynein heavy chain 1, axonemal	3.00E‐14	EKC41830.1	*Crassostrea gigas*
3235	PREDICTED: LOW QUALITY PROTEIN: Spectrin beta chain‐like	9.00E‐09	XP_013069807.1	*Biomphalaria glabrat*
3893	PREDICTED: Phosphoenolpyruvate carboxykinase GTP‐like	2.00E‐12	XP_005103800.1	*Aplysia californica*
5203	PREDICTED: Probable G protein‐coupled receptor 139	4.00E‐09	XP_005104219.1	*Aplysia californica*

### 3.4 Sharing of outlier loci

Consistent with our prediction, we observed high levels of shared divergent outliers between the closer Corrubedo and Silleiro sites, but low levels of shared genomic divergence with the northern Burela site (Table 3). Of 193 divergent outlier SNPs identified using at least two outlier detection methods, 6.2% of these loci were shared among all three sites. We observed a much greater degree of allele sharing of divergent loci between Corrubedo and Silleiro, with 34.4% of loci shared, relative to sharing between either Burela and Corrubedo or Burela and Silleiro (9.9%–15.1%). Sharing of loci exhibiting potential balancing selection was low across all comparisons (1%–2%).

**Table 3:** Total number of unique divergent outlier SNP loci identified in *F*_ST_ outlier tests between different tidal levels in each site significant at 5% after SGoF correction of FDIST outliers and false discovery rate correction of BayeScan and pcadapt outliers, and number and percentage of divergent outlier loci shared between compared sites

Outlier detection method	Sites compared	Total unique *F*_ST_ outlier loci identified	Percentage and number of shared loci
FDIST	All	240	5.8% (14)
	Burela–Corrubedo	203	13.3% (27)
	Burela–Silleiro	192	11.5% (22)
	Corrubedo–Silleiro	149	30.2% (45)
BayeScan	All	59	6.8% (4)
	Burela–Corrubedo	48	14.6% (7)
	Burela–Silleiro	49	12.2% (6)
	Corrubedo–Silleiro	40	35.0% (14)
pcadapt	All	275	4.4% (12)
	Burela–Corrubedo	242	11.1% (27)
	Burela–Silleiro	192	8.3% (16)
	Corrubedo–Silleiro	183	26.2% (48)

We compared our observations of genomic parallelism with randomly resampled loci to determine whether greater sharing occurred than by chance. All pairwise comparisons exceeded the 95% confidence interval of random sharing between two sites (3.8%–4.6%) and three sites (0.19%–0.23%). To account for random sharing of loci caused by non‐independence of southern populations, we also compared our data with simulations conducted by Westram et al., 2014. Our estimates of shared divergence between Corrubedo and Silleiro (34.4%) are more than double those expected by chance at 95% significance (8% shared outlier loci) between populations with even the highest simulated migration rates (10^−6^) between sites. *F*_ST_ values for the same ecotypes between simulated sites with high connectivity are also substantially lower (0.0541–0.0679) than observed in this study (0.141–0.211), indicating that much greater levels of connectivity would be required to generate observed patterns of genomic parallelism by chance.

### 3.5 Linkage disequilibrium

We observed a low level of potential physical linkage among SNPs in our dataset. Of the 4,256 SNPs, 537 (12.6%) exhibited elevated linkage (*r* ^2^ > 0.2) in all sites, whereas 36 divergent loci exhibited linkage (18.7%), indicating slightly greater linkage of outlier loci relative to the genomic average. Linkage between outliers did not appear to substantially influence rates of outlier sharing. Among loci exhibiting no linkage, 34.3% were shared between Corrubedo and Silleiro, 15.1% between Burela and Corrubedo, 10.0% between Burela and Silleiro, and 5.7% shared overall.

### 3.6 Population structure and admixture

**Figure 2:**
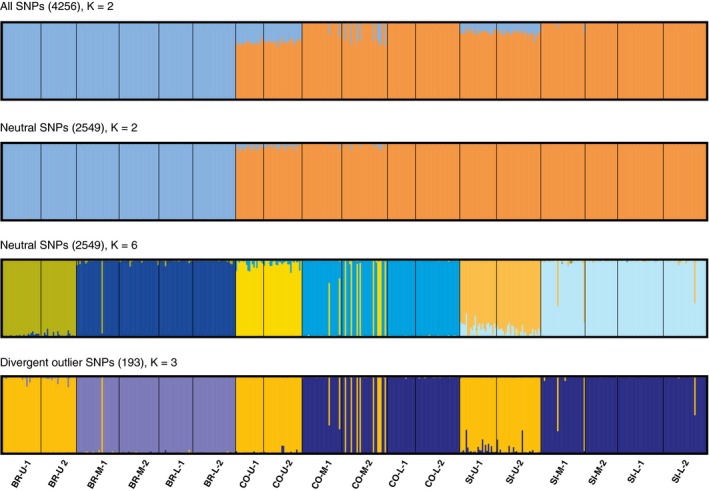
STRUCTURE results for all sets of loci, with admixture proportions for *K* source population simulations summarized over 3 runs using CLUMPAK, for all SNPs (*K* = 2), neutral SNPs (*K* = 2, *K* = 6), and divergent SNPs (*K* = 3), at three tidal levels, upper shore (U), lower shore (L), mid‐shore (M) in three sites, Burela (BR), Corrubedo (CO), Silleiro (SI)

STRUCTURE analyses showed low levels of admixture within phenotypically intermediate mid‐shore individuals across sites. To quantify admixture among ecotypes and sites, we also explored admixture rates for *K* = 6, as multiple biologically meaningful values of *K* may exist for a dataset (Wang et al., 2007). We observed low levels of admixture and predominantly wave ecotype genetic background in mid‐shore locations in all three sites when comparing neutral loci at *K* = 6 (Figure 2). We identified only three phenotypically intermediate mid‐shore individuals with *Q* values reflecting potential recent hybridization or backcrosses (*Q* = 20%–80%, Figure 2).

**Figure 3:**
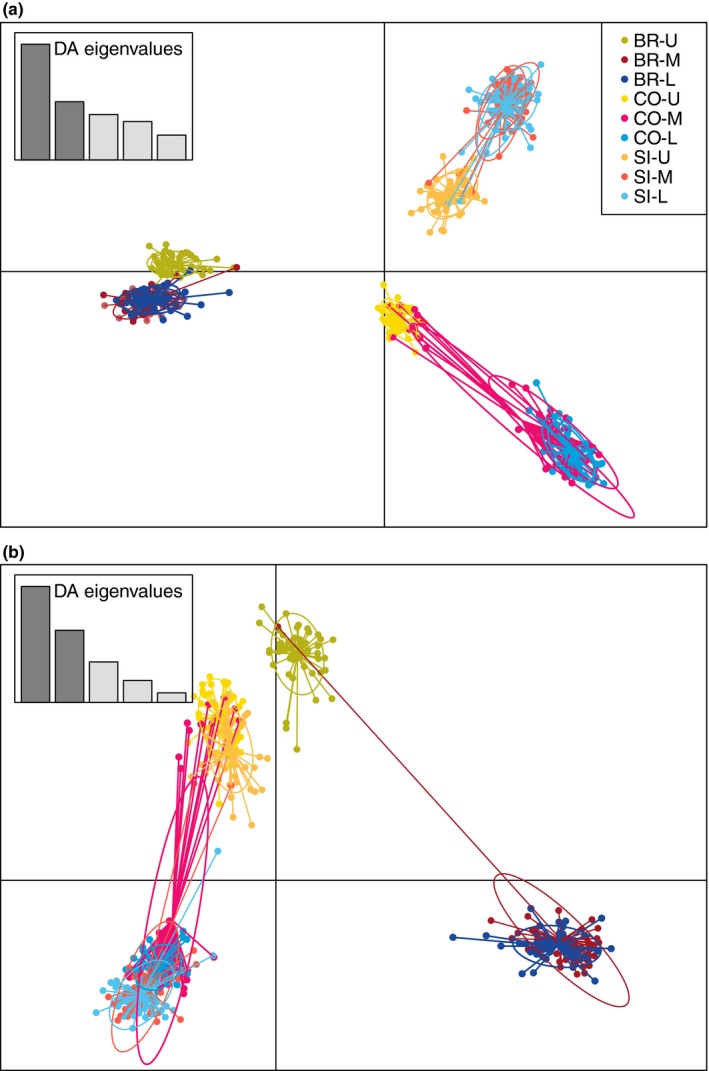
Discriminant analysis of principal components conducted on (a) 2,549 neutral loci and (b) 193 outlier loci in Burela (BR), Corrubedo (CO), and Silleiro (SI) upper shore (U), mid‐shore (M), and lower shore (L) individuals

**Figure 4:**
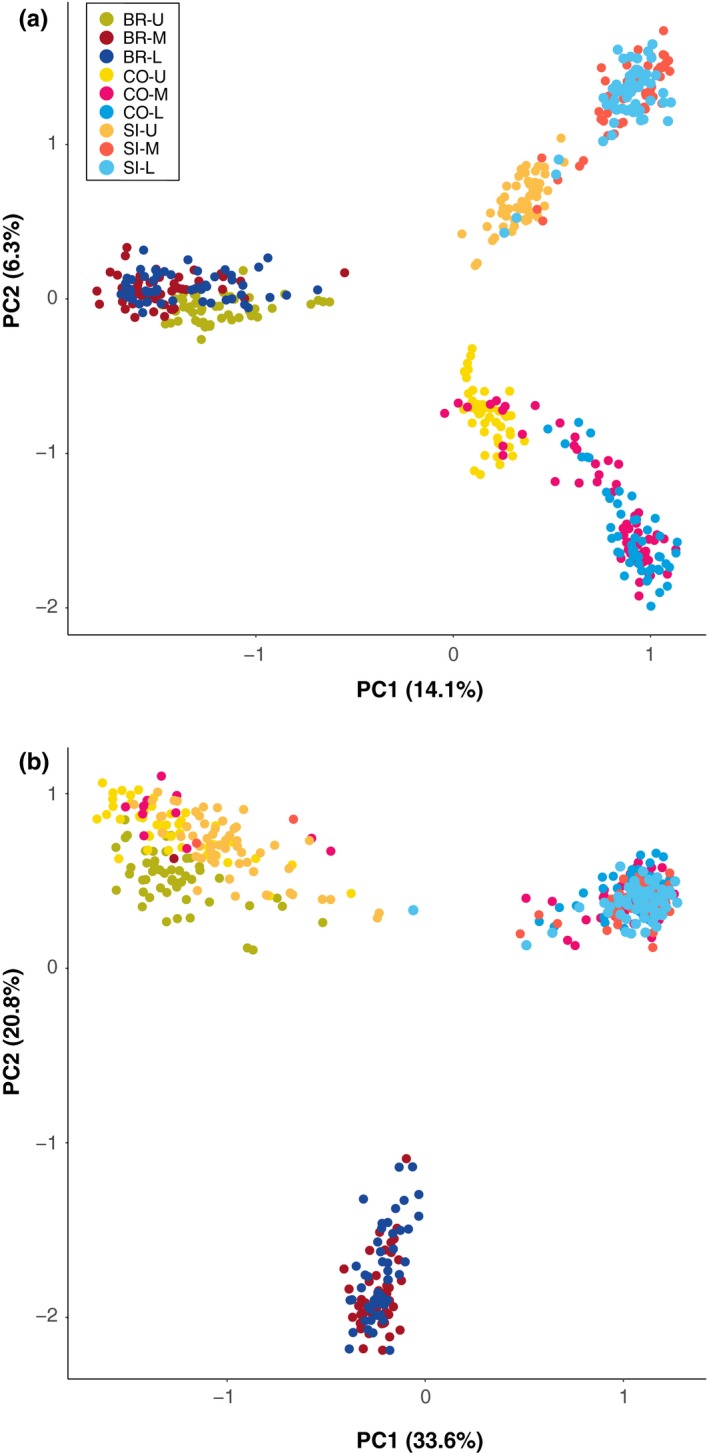
Plotting of first two principal component axes calculated from (a) 2,549 neutral loci and (b) 193 outlier loci in Burela (BR), Corrubedo (CO), and Silleiro (SI) upper shore (U), mid‐shore (M), and lower shore (L) individuals

Population clustering revealed clear separation between glacial refugia (Figure 2). When including all 4,256 loci for cluster identification, Δ*K* was maximized at *K* = 2, with both tidal level samples in Burela forming one cluster, and all tidal levels in Silleiro and Corrubedo forming the second cluster, although upper shore individuals in these localities also shared a small proportion of ancestry with the Burela cluster (Figure 2). Clustering of 2,549 neutral loci also identified two distinct clusters corresponding to glacial refugia. DAPC using neutral loci indicated support for *K* = 6 clusters (Figure 3). These clusters corresponded to wave and crab ecotype genetic backgrounds distinct to each site, with individuals of intermediate phenotype clustered with wave ecotype individuals. PCA of neutral loci supported these observations, as six distinct clusters that separated glacial refugia, sites, and ecotypes were observed when plotting individual scores on the first two PC axes (Figure 4).

Population clustering of all 193 divergent loci indicated shared clustering of crab ecotypes, but separate genetic backgrounds for wave ecotypes between glacial refuges. We identified Δ*K* maximum at *K* = 3, with upper shore individuals from Burela, Corrubedo, and Silleiro clustering together, while lower and mid‐shore individuals from Corrubedo and Silleiro formed a distinct cluster separate from mid and lower shore individuals from Burela. Similarly, DAPC of divergent loci identified six clusters in three larger groupings, corresponding to separation between Burela lower shore individuals and lower shore individuals from Corrubedo and Silleiro, and a third cluster of upper shore individuals from all sites (Figure 3). PCA of divergent loci also identified maximum separation between all crab ecotypes in one cluster and separation of distinct groups between southern and northern wave ecotypes (Figure 4).

### 3.7 Genetic differentiation

Quantification of genetic differentiation revealed strong roles for both site and tidal level in partitioning genetic variation. In AMOVA conducted with all SNPs, we observed greatest differentiation between site subgroups (*F*_SC_ = 0.219), indicating that geography is the primary level of differentiation among sites or tidal levels (Table 4). However, when Burela was excluded from analysis, the greatest differentiation was instead observed between the crab and wave ecotypes collected from the upper and lower tidal levels, respectively (*F*_CT_ = 0.147). Among neutral loci, the greatest source of genetic differentiation was observed between geographic subgroups (*F*_SC_ = 0.20). This pattern of differentiation was similar in comparisons of Corrubedo and Silleiro, but differences between the ecotypes from the upper and lower tidal level also contributed to a nearly equal degree to genetic divergence (Table 4). Comparisons of divergent outlier loci showed very high differentiation between sites and also between ecotypes collected from the upper and lower tidal levels. However, differentiation driven by tidal level predominated in analyses restricted to Corrubedo and Silleiro. Pairwise comparisons of *F*_ST_ for neutral and divergent loci between transects, sites, and tidal levels revealed geographic differentiation driven by divergence with the Burela site, and nonsignificant differentiation between transects on the same tidal level within sites (Supporting Information Table S1 and S2).

**Table 4:** Analysis of molecular variance (AMOVA) results for all loci, neutral loci, or divergent loci grouped by site or tidal level for three sites and for Silleiro and Corrubedo only

Marker set	Number of compared loci	Populations compared	Grouped by	*F*_CT_	*F*_SC_
All SNP loci	4253	All	Site	0.152	0.16
			Tidal level	0.086	0.219
	3455	Corrubedo–Silleiro	Site	0.072	0.166
			Tidal level	0.147	0.123
All neutral loci	2547	All	Site	0.154	0.110
			Tidal level	0.031	0.200
	1911	Corrubedo–Silleiro	Site	0.090	0.117
			Tidal level	0.073	0.126
All divergent loci	193	All	Site	−0.175	0.662
			Tidal level	0.364	0.484
	177	Corrubedo–Silleiro	Site	−0.371	0.660
			Tidal level	0.542	0.259

### 3.8 Phylogenetic analysis

**Figure 5:**
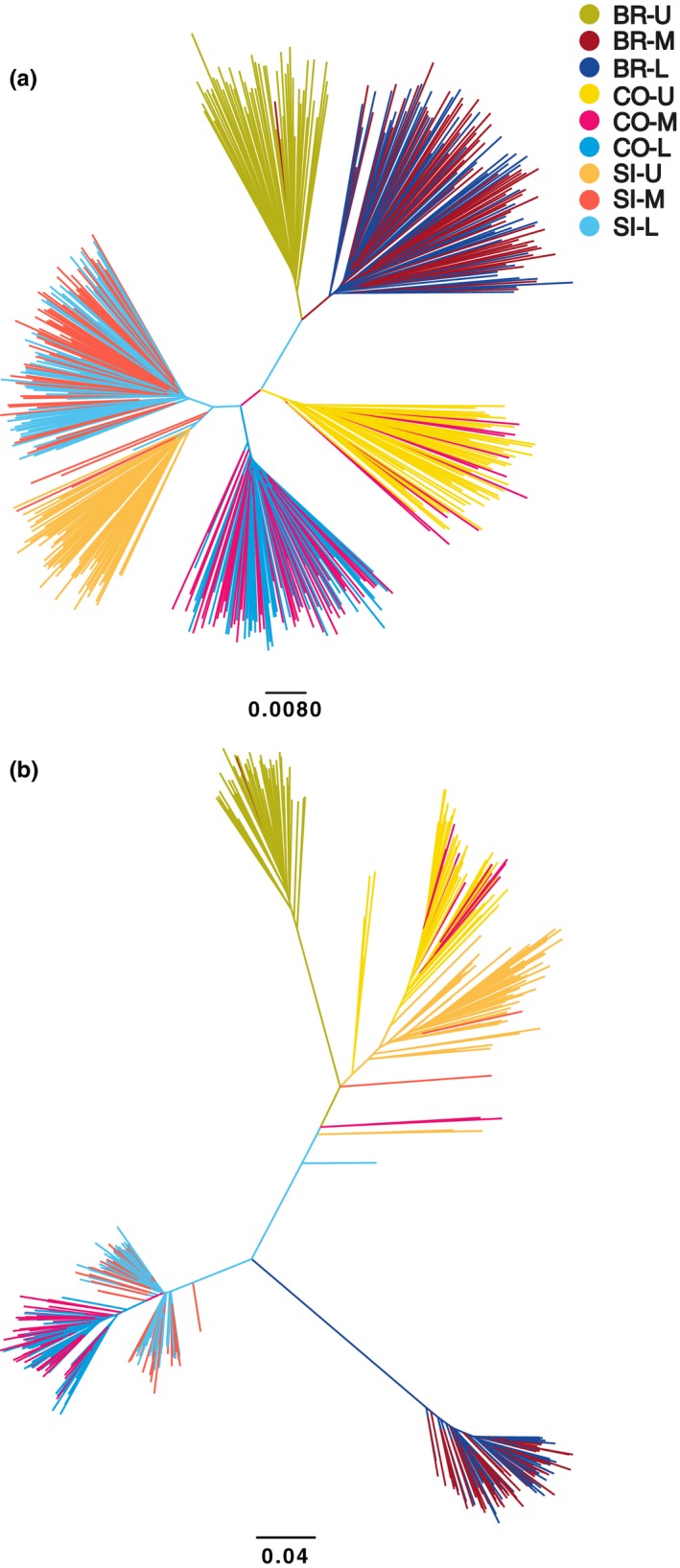
Individual neighbor‐joining trees of Cavalli‐Sforza and Edwards' chord distances calculated from (a) 2,000 neutral loci and (b) 193 outlier loci in Burela (BR), Corrubedo (CO), and Silleiro (SI) upper shore (U), mid‐shore (M), and lower shore (L) individuals

SNP phylogenies indicated evolutionary independence of randomly selected neutral loci between sites and ecotypes, and independence of divergent loci between northern and southern sites. Individual neutral phylogenies revealed six distinct branches corresponding to upper and lower shore individuals within each site (Figure 5). Divergent loci produced a different tree topology, with clear separation between lower shore individuals between northern and southern sites, and distinct but clustered branches for upper shore individuals across sites, consistent with results from population structure analyses (Figure 5).

## 4 DISCUSSION

Population genetic analysis of RAD‐derived SNPs sequenced in *L. saxatilis* upper, lower, and mid‐shore samples from three separate sites in Spain revealed substantial levels of genomic parallelism of loci divergent between sampled tidal levels that declined when compared with a distant site colonized from a separate glacial refuge. These results suggest that the opportunity for historical or contemporary gene flow influences levels of parallel differentiation at the genomic level.

### 4.1 Parallelism of divergent loci

High levels of shared divergence observed between samples from the same tidal levels in Silleiro and Corrubedo compared with Burela support a role for historical or contemporary separation in affecting levels of genomic parallelism. Genomic regions potentially under divergent selection were largely non‐parallel between Burela and Silleiro or Burela and Corrubedo. In contrast, a large proportion (34.4%) of the total identified divergent outlier sites were shared between Silleiro and Corrubedo, even though the latter two sites were significantly differentiated for neutral loci (*F*_SC_ = 0.126), and phylogenetically distinct. These results differ from those observed in *L. saxatilis* studies by Ravinet et al. (2016) and by Westram et al. (2014). In these studies, outlier loci sharing between the same ecotypes across the entire eastern Atlantic range did not differ strongly by geographic separation or level of genomic differentiation, and no increase in parallelism was observed even when spatial scale was restricted to ecotype comparisons between geographically close sites in Sweden.

The timing of adaptive divergence of ecotypes following colonization from separate glacial refugia may explain differences between parallelism observed in this study and past studies in other parts of Europe. The Spanish *L. saxatilis* ecotype pairs are believed to have colonized multiple shores following emergence from two distinct glacial refugia, with southern populations emerging approximately 40,000 years before present, and northern ecotypes emerging more recently. In contrast, Swedish and UK ecotype pairs are believed to have emerged in the past 10,000 years (Butlin et al., 2014; Johannesson et al., 2010). Extended time for divergence of Spanish ecotype pairs from southern localities (Corrubedo and Silleiro) may have provided greater opportunity for adaptive mutations to accumulate, migrate, and be acted upon by selection in geographically close sites, increasing levels of observed genomic parallelism at these locations. In this scenario, highly differentiated genomic regions shared between *L. saxatilis* ecotypes may represent strongly selected variation introduced through migration from other localities, indicating an instance of “evolution in concert” (Johannesson et al., 2010). Alternatively, these shared loci may reflect the small complement of shared ancestral standing variation within the southern glacial refuge that enabled rapid adaptive divergence of ecotypes across multiple colonizations (Barrett & Schluter, 2008; Johannesson et al., 2010). Our results also differ from the observed high parallelism of capture‐sequenced outlier loci across glacial refuges identified by Westram et al., 2016. This difference could be attributed to sampling bias produced by outlier selection. However, weaker differentiation due to recent introduction of loci from different refugia, or different epistatic interactions of shared standing variation across sites, reducing time of selection or local allelic fitness may also be possible.

Multiple explanations also exist for the largely non‐parallel observed patterns of total genomic divergence within ecotype pairs in Burela relative to the other sites. Due to geographic distance, Burela ecotypes would be expected to experience less frequent introduction of alleles from Corrubedo or Silleiro and vice versa, and this reduction in adaptive admixture will decrease the extent of shared genomic divergence between ecotype pairs. Comparison of genetic structure of *L. saxatilis* populations across northwestern Spain has also revealed a potential biogeographic barrier between northern and southern populations corresponding to variation in climatic and oceanographic features that may limit dispersal between sites in the present study (Piñeira, Quesada, Rolán‐Alvarez, & Caballero, 2008). The presence of multiple refugia increases the recent evolutionary independence of northern and southern sites, such that genetic backgrounds of divergent traits may have continued to evolve through periods of isolation to be less suitable for the establishment of introduced alleles from distant sites (Conte et al., 2012). Even without genetic barriers to adaptive gene flow or variation in divergent traits between sites, many shared ecologically relevant traits in ecotypes may possess a polygenic inheritance or may arise from many overlapping pathways to produce similar phenotypes, increasing the probability that alternative alleles may be selected (Hoekstra & Nachman, 2003; McGee, Neches, & Seehausen, 2016). Crosses between individuals of similar ecotype from multiple sites may be useful in determining the genetic architecture of traits divergent between crab and wave ecotypes, and in revealing whether negative epistasis between alleles from different genetic backgrounds has played a role in limiting observed levels of genomic parallelism.

Our results show marked difference from other studies of parallel genomic divergence that emphasize a role for standing variation in driving local adaptation (Barrett & Schluter, 2008; Nelson & Cresko, 2018; Schluter & Conte, 2009). Interestingly, divergent loci exhibited clear separation between refugia in wave ecotypes, whereas shared phylogenetic grouping and population structure clustering were observed for these loci in crab ecotypes. These results suggest that shared variation has produced crab ecotypes, whereas distinct complements of adaptive alleles led to wave ecotype formation. The low overlap of outliers between glacial lineages in *L. saxatilis* suggests that divergent adaptation to intertidal conditions may be achieved without drawing on species‐level ancestral variation. Although separate complements of standing variation that arose after separation of northern and southern sites may have enabled local ecotype formation, our results demonstrate that ancient variation may not be critical for rapid adaptation. These results are contrary to observation in a highly connected model system, marine and freshwater sticklebacks, in which ancient alleles exhibiting high differentiation are shared between replicate populations across a large range (Nelson & Cresko, 2018). This difference may be attributed to greater levels of connectivity between stickleback populations, allowing regular movement of adaptive variation to geographically distant sites, and ensuring a range‐wide pool of genomic diversity (Schluter & Conte, 2009). In contrast to another model system, our observation of high levels of shared divergence between close sites from the same refuge differs from results observed in stick insects, in which low sharing of divergent regions was identified (Soria‐Carrasco et al., 2014). Differing stages of speciation between the *L. saxatilis* and *Timema* systems may account for observed differences in genomic parallelism. Extended divergence time between ecotypes in southern populations may have allowed opportunity for chance introduction of favorable alleles between populations. Examples of such chance events include the introduction of alleles adaptive at high altitude from Denisovan ancestors in human populations in Tibet (Huerta‐Sánchez et al., 2014), and the initiation of speciation by introduction of an adaptive beak morphology allele in Darwin's finches (Lamichhaney et al., 2018). Thus, the temporal context of parallel adaptation may also play a role in dictating genomic parallelism by increasing the timeframe during which these events may occur. These hypotheses may be tested in *L. saxatilis* ecotypes through characterization of molecular diversity within divergent genomic regions and adjacent sites using whole genome sequence. These analyses, as have recently been conducted in stickleback marine and freshwater ecotypes (Nelson & Cresko, 2018), could allow differentiation between hypotheses positing that selection on ancestrally shared standing variation has produced shared regions of local divergence between ecotypes, or that shared genomic divergence has instead occurred through repeated selective sweeps of the same *de novo* mutation shared through infrequent migration.

While many adaptive explanations for patterns of genomic divergence exist, caution is needed in attributing adaptive mechanisms underlying observed genomic differentiation identified solely through genome scans. Identification of *F*_ST_ outliers should be treated as preliminary statistical observations of potential targets of selection and cannot be used on their own to differentiate adaptive from neutral hypotheses. Several alternative explanations contingent on demography or localized features of genome structure also exist for observations of regions of increased divergence and have been covered in recent discussions of the limitations of genome scans (see Bierne, Roze, & Welch, 2013; Bierne, Welch, Loire, Bonhomme, & David, 2011; Ravinet et al., 2017; Wolf & Ellegren, 2017). Greater confidence that identified divergent regions also contribute to ecologically relevant traits may be achieved through association of those regions with phenotypic and environmental variables, and through molecular characterization of those regions or linked loci.

Here, we annotated a very small proportion of the *F*_ST_ outliers that exhibit functions that may be important in ecotype divergence and speciation (~4%) (Table 2). One of the outliers, spondin‐1‐like protein, has also been detected between *L. saxatilis* ecotypes from populations in Britain (Westram et al., 2016). This type of protein has been detected as a candidate for shell formation genes in pearl oyster and may play a similar role in ecotype shell architecture (Kinoshita et al., 2011). Moreover, vertebrate SCO‐spondin proteins have been regarded as similar to invertebrate gel‐forming mucins (Lang et al., 2016) which could also be involved in shell formation and that have also been identified in a previous transcriptome scan in *L. saxatilis* (Galindo, Grahame, & Butlin, 2010). Two other annotated outliers, axonemal dynein heavy chain and G protein‐coupled receptor, have been detected as outliers in Swedish *L. saxatilis* populations (Ravinet et al., 2016). Axonemal dynein heavy chain could be involved in flagellar movement in sperm and therefore linked to speciation (Carvalho, Lazzaro, & Clark, 2000). G protein‐coupled receptors could also be related to adaptation like the case of MC1R in vertebrates (Hoekstra, Hirschmann, & Bundey, 2006). Characterization of the function of these outlier loci across multiple instances of ecotype formation is a clear objective for future follow‐up studies.

### 4.2 Population genetic structure and admixture

Population clustering with RAD‐derived SNPs enabled separation of distinct site and tidal level groups with minimal admixture using neutral loci, although the presence of separate glacial refugia dominated the clustering pattern observed in STRUCTURE comparisons. However, the overall pattern of divergence across measurements of population structure, individual phylogenetic separation, and differentiation indicates the presence of geographically distinct crab and wave ecotype pairs within each site.

We detected much less frequent hybridization between ecotypes than previously identified, as well as greater genetic separation between both tidal level and sites (see Galindo et al., 2013). Greater precision achieved using a larger set of codominant SNP markers to detect differentiation and admixture could partially account for lower observed amounts of admixture not detected by AFLP markers. Similar improved resolution of population structure using SNPs has revealed greater levels of genomic differentiation than inferred from previous AFLP studies in phenotypically distinct cichlid fish species (Keller et al., 2013), as well as in delineating genetically distinct groups in Wilson's warblers (*Cardelina pusilla)* (Ruegg et al., 2014).

Alternatively, intermediate phenotype *L. saxatilis* in this study may result from phenotypic plasticity. Previous studies of shell development have found evidence of plastic changes in littorinid shell ridging depending on developmental environment (Boulding, Buckland‐Nicks, & Van Alstyne, 1993). However, laboratory‐rearing experiments show that plastic variation in shell phenotype within *L. saxatilis* ecotypes is much less than the genetic variation between the crab and wave ecotypes (Conde‐Padín, Caballero, & Rolán‐Alvarez, 2009; Hollander & Butlin, 2010; Saura et al., 2012).

A third hypothesis is that alleles underlying banding and ridging are present in wave ecotypes as standing variation. This hypothesis may be investigated by determining the genetic architecture underlying these traits in each ecotype, using genome‐wide association mapping.

Lastly, fluctuation in levels of admixture observed in this study compared to the study conducted by Galindo et al. (2013) may support variation in temporal and spatial exogenous barriers to gene flow Galindo et al., 2014. Phenotypically intermediate individuals may still represent individuals of mixed genetic background, but hybridization between ecotypes may have occurred several generations ago, allowing time for selection against the crab ecotype genetic background. Future studies seeking to clarify barriers to gene flow could be conducted by comparing phenotypic and genetic composition of intermediate mid‐shore individuals across varying microhabitats and seasons.

### 4.3 Genetic differentiation and phylogenetic analysis

Consistent with observed infrequent admixture and clear genetic clustering of tidal levels, comparisons of sources of genomic differentiation revealed more extensive genetic differentiation between each tidal level than has been previously observed (Galindo et al., 2009; Rolán‐Alvarez et al., 2004). Similarly, measurement of neutral *F*_ST_ (0.17) in this study was substantially larger than values observed in comparisons between ecotypes using AFLP markers (*F*_ST_ = 0.064; Galindo et al., 2009). Similar levels of divergence have been observed in comparisons between sympatric Lake Victoria cichlids (Keller et al., 2013), whereas slightly greater levels of overall divergence have been observed between sympatric *Heliconius* host races (Martin et al., 2013). This observed level of baseline genomic differentiation suggests that progression toward speciation between ecotypes in upper and lower tidal levels may be more extensive than previously believed, potentially to an early stage of genome‐wide isolation (Feder, Egan, & Nosil, 2012; Seehausen et al., 2014). However, although current results indicate strong isolation and genomic divergence, sampling of RAD markers may not perfectly reflect genomic distribution of divergent sites and corresponding evolutionary history. Future comparison of whole genome sequence from individuals in either tidal level will help clarify the degree of genomic isolation and progress toward speciation between *L. saxatilis* ecotypes.

Our phylogenetic and genetic differentiation analyses of neutral loci suggest a large degree of genetic independence of ecotypes in each site. Repeated observation of mitochondrial phylogenies that cluster by sampling site rather than ecotype, including between southern sites (Quesada et al., 2007; Tirado et al., 2016), and support for independent ecotype origin between northern and southern sites using demographic modeling (Butlin et al., 2014), together provide strong evidence for local emergence of ecotype pairs. Although this scenario cannot be completely rejected between southern sites, the observation of separate neutral SNP phylogenies in the present study and the occurrence of high between‐ecotype levels of divergence that nearly match levels of distance driven isolation provide evidence against ongoing gene flow between ecotypes eroding historical patterns of divergence. Additionally, recent simulations of divergence with gene flow suggest that unless high levels of between‐ecotype migration occur, phylogenetic methods should be able to differentiate between local divergence and secondary contact (Pérez‐Pereira, Quesada, & Caballero, 2017). Thus, our results provide additional support for local origin of ecotypes across sites, but future whole genome studies may more explicitly test this hypothesis through demographic reconstruction with coalescent models.

## 5 CONCLUSIONS

In the present study, we identified substantial sharing of total divergent outlier loci between upper and lower shore ecotypes across two geographically close sites that was not observed at a third distant site colonized from a separate glacial refuge. These results support the hypothesis that ancestral or contemporary connectivity of populations may play a role in dictating genomic parallelism. These results also indicate that shared ancestral variation may not be necessary for ecological adaptation and divergence.

Additionally, we identified very little admixture between upper and lower shore ecotypes, despite the presence of phenotypically intermediate individuals found at the mid‐shore. Lastly, we identified a greater role for differentiation between tidal levels contributing to genetic variation than was found in previous studies, potentially enabled by greater genomic sampling. Taken together, these results indicate greater progression of *L. saxatilis* ecotypes along a continuum of speciation toward genomic isolation than has been previously uncovered for this model system.

## CONFLICT OF INTEREST

None declared.

## AUTHOR CONTRIBUTIONS

T.K. designed the project, conducted laboratory work and statistical analysis, and wrote the manuscript. J.G. helped design the project, collected samples, conducted statistical analysis, and helped write the manuscript. E.G.B. helped design the project, supervised the laboratory work, assisted with interpretation of the statistical analysis, and helped write the manuscript.

## DATA ACCESSIBILITY

Stacks consensus assembly, vcf and haplotype files, STRUCTURE input and results files, genepop files for all loci, neutral loci, and identified divergent outlier loci from BayeScan, *pcadapt*, and FDIST, and individual sample metadata available from the Dryad Digital Repository: https://doi.org/10.5061/dryad.8n2b32q. Raw reads will be uploaded to the NCBI short‐read archive with submission number SUB4134175.
